# Cichlids and stingrays can add and subtract ‘one’ in the number space from one to five

**DOI:** 10.1038/s41598-022-07552-2

**Published:** 2022-03-31

**Authors:** V. Schluessel, N. Kreuter, I. M. Gosemann, E. Schmidt

**Affiliations:** Institute of Zoology, University of Bonn, Meckenheimer Allee 169, Poppelsdorfer Schloss, 53115 Bonn, Germany

**Keywords:** Zoology, Animal behaviour, Cognitive neuroscience

## Abstract

The numerical understanding of cichlids and stingrays was examined regarding addition and subtraction abilities within the number space of one to five. Experiments were conducted as two-alternative forced-choice experiments, using a delayed matching to sample technique. On each trial, fish had to perform either an addition or subtraction, based on the presentation of two-dimensional objects in two distinct colors, with the color signaling a particular arithmetic process. Six cichlids and four stingrays successfully completed training and recognized specific colors as symbols for addition and subtraction. Cichlids needed more sessions than stingrays to reach the learning criterion. Transfer tests showed that learning was independent of straightforward symbol memorization. Individuals did not just learn to pick the highest or lowest number presented based on the respective color; instead, learning was specific to adding or subtracting ‘one’. Although group results were significant for both species in all tests, individual results varied. Addition was learned more easily than subtraction by both species. While cichlids learned faster than stingrays, and more cichlids than stingrays learned the task, individual performance of stingrays exceeded that of cichlids. Previous studies have provided ample evidence that fish have numerical abilities on par with those of other vertebrate and invertebrate species tested, a result that is further supported by the findings of the current study.

## Introduction

Quantity discrimination abilities have been demonstrated in all vertebrate classes as well as several invertebrate species^1–4^. This seems reasonable, as quantity discriminations (including numerical discriminations) are vital for many species in the context of daily activities such as foraging^5,6^, predator avoidance^7^, schooling and mate choice^8,9^. Quantity discrimination can be based on several parameters such as continuous (e.g. size or surface area covered by items) or discrete variables (number of items). While both strategies are not mutually exclusive and may, when combined, yield a more successful outcome^1^, some species use only continuous variables such as size to base their choice on and seem unable to use numerical information by itself^10^.

Several studies have proposed that there are two different systems for numerical differentiation^11–15^. However, there is an ongoing discussion within the literature on whether one or two systems exist that are utilized for numerical discrimination. Most studies on fish support the two-system hypothesis^16^. The current study did not investigate this issue further and results are discussed in the context of the findings by the aforementioned study^16^. With the first of the two hypothesized systems, the ‘object file system’ (OFS), small amounts, differing in only one item, can be recorded precisely^6^; however, the upper limit is low and ranges between 3 and 5 items for most individuals^17,18^. For the OFS, the numerical ratio is irrelevant; instead, the overall image composition or the quantity of items stored in the working memory is essential^19^. With the help of the second system for numerical discrimination, the ‘analogue magnitude system’ (AMS), larger numerical values can be roughly estimated and compared^12^. The precision of the size distinction is subject to Weber's law and depends on the relationship between the two quantities to be discriminated^17,20,21^. Discrimination is more successful with an increasing ratio between the two numerical samples.

In order to examine these two proposed systems and their limitations, studies have been carried out on a variety of species, such as primates^22^, salamander (*Plethodon cinereus*)^6^, mockingbird (*Mimus polyglottos*)^23^, jungle crows (*Corvus macrorhynchos*)^24^, sticklebacks (*Gasterosteus aculeatus*)^25^, guppies (*Poecilia reticulata*)^26^ or the eastern mosquito fish (*Gambusia holbrooki*)^9^. Most of these studies found upper limits for the OFS around 3–5, with a distinction between either 3:4 or 4:5 items not being successful anymore. However, guppies still successfully discriminated 4:5 and sticklebacks even 6:7 items^25,26^. In freshwater stingrays (*Potamotrygon motoro*) and bamboo sharks (*Chiloscyllium griseum*) the OFS was recently shown to be 4, with 4:3 and 5:3 being discriminated successfully by stingrays and sharks respectively, while 4:5 was not^27^. In cichlids, the OFS appears to range from 1 to 5, with 4:5 still being discriminated successfully by most individuals^28,29^. While not all studies support the presence of both an OFS and AMS in all species—in fact, only honeybees, within the invertebrates tested so far, were able to discriminate numerical quantities greater than four^30,31^—studies on fish largely suggest the co-existence of two such systems^16^. Aside from cardinal information, numerical abilities can also include ordinal competence, which has been investigated in non-human species^32–36^, including fish^37^.

Whether vertebrates other than humans and primates can solve more complex numerical tasks or arithmetic problems such as addition and subtraction is—despite some promising studies—currently still unclear^38–40^. Accordingly, within the range of the OFS, simple mathematical calculations, such as addition and subtraction, have only been investigated in a few species such as primates (chimpanzees^41–43^, orangutans^44^, rhesus monkeys^45^ and vervet monkeys^46^), birds (gray parrot^47,48^, pigeons^49^ and chicks^50^), as well as spiders^51,52^ and honey bees^53^.

In the most recent study^53^, honeybees recognized colors as symbols for addition and subtraction tasks. The bees successfully added and subtracted objects and applied this knowledge in transfer tests to an unknown number of objects, indicating acquisition of long-term rules and short-term working memory. First, the bees were taught to fly into a Y-shaped sample room, in which they were initially presented with a single visual ‘test’ stimulus (blue or yellow) displaying a certain number of geometric objects. The animals then had to fly through an opening into the decision chamber and select one of two choice stimuli (stimuli A and B). These contained one element more (stimulus A) and one element less (stimulus B) than the test stimulus shown previously. Depending on the color of the test stimulus, the bees successfully performed additions and subtractions within the number space of 1 to 5.

Based on the design by Howard et al.^53^, the numerical abilities of cichlids and freshwater stingrays were tested in the current study. More general numerical abilities of stingrays and bamboo sharks had only recently been investigated for the first time in elasmobranchs^25^ and found to match those of teleost species. Previous studies have demonstrated that both cichlids and stingrays are well suited for cognition experiments, displaying a wide range of cognitive abilities, ranging from visual discrimination experiments to spatial orientation^54–62^.

## Materials and methods

### Animals

Experimental animals were eight cichlids (*Pseudotropheus zebra*), obtained from a commercial dealer (Merz, Germany) and eight experimentally naïve freshwater stingrays (*Potamotrygon motoro*) from Frankfurt Zoo (Germany). Six of the cichlids had previously participated in cognition experiments, the other two were experimentally naïve. Animals were subjected to a natural light–dark cycle and experiments were conducted in the morning and early afternoon six days per week. Food was only provided during the experiments and consisted of cichlid food pellets (Sera Granugreen) and of earthworm, shrimp or mussel for the stingrays.

### Set up

**Figure 1:**
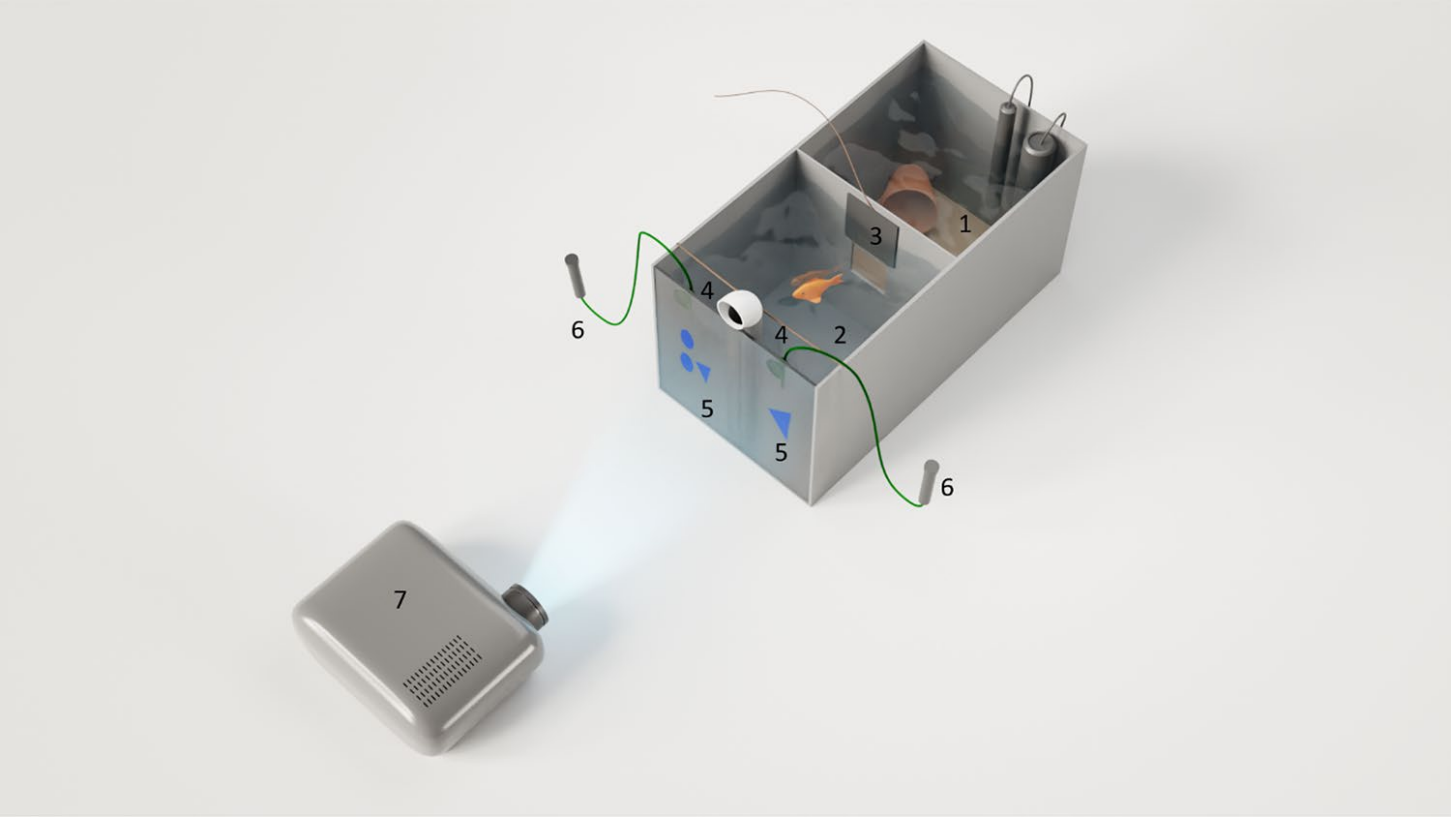
Experimental setup for the cichlids. 1) start box, 2) experimental area, 3) guillotine door, 4) decision areas, 5) stimuli, 6) feeding tubes, 7) projector.

For both species, housing tanks also served as experimental tanks. Cichlids were kept individually in 61 cm × 31 cm × 31 cm test tanks with a maximum volume of 54 L (Fig. 1). Individual tanks were halved by a gray, opaque partition wall into a front and a rear compartment. The fish could swim through a 7 cm × 7 cm window (operated manually by a transparent guillotine door) in the partition to use both areas. The floor of the living area (starting compartment) was covered with sand, while it was empty in the front compartment (experimental area). In the living area, a clay pot served as a hiding place; additionally, it contained a plant, a pump and an aquarium heater (~ 25 °C). The water quality was maintained by weekly water changes. A Plexiglas divider was placed in the middle of the milky-colored front of the tank, and a line was provided 5 cm before the front, as to divide the area into two equally large compartments (decision areas). Using a projector (Optoma), PowerPoint slides were presented on the milky-colored front, featuring two-dimensional geometric symbols as stimuli. Using pipettes attached to long tubing, one food pellet was expelled immediately once a correct choice was made. The tubes were fixed with suction cups to the front wall and situated right above the stimulus projections. All tests were observed via a camera (Logitech), placed on a plastic rope above the set-up.

**Figure 2:**
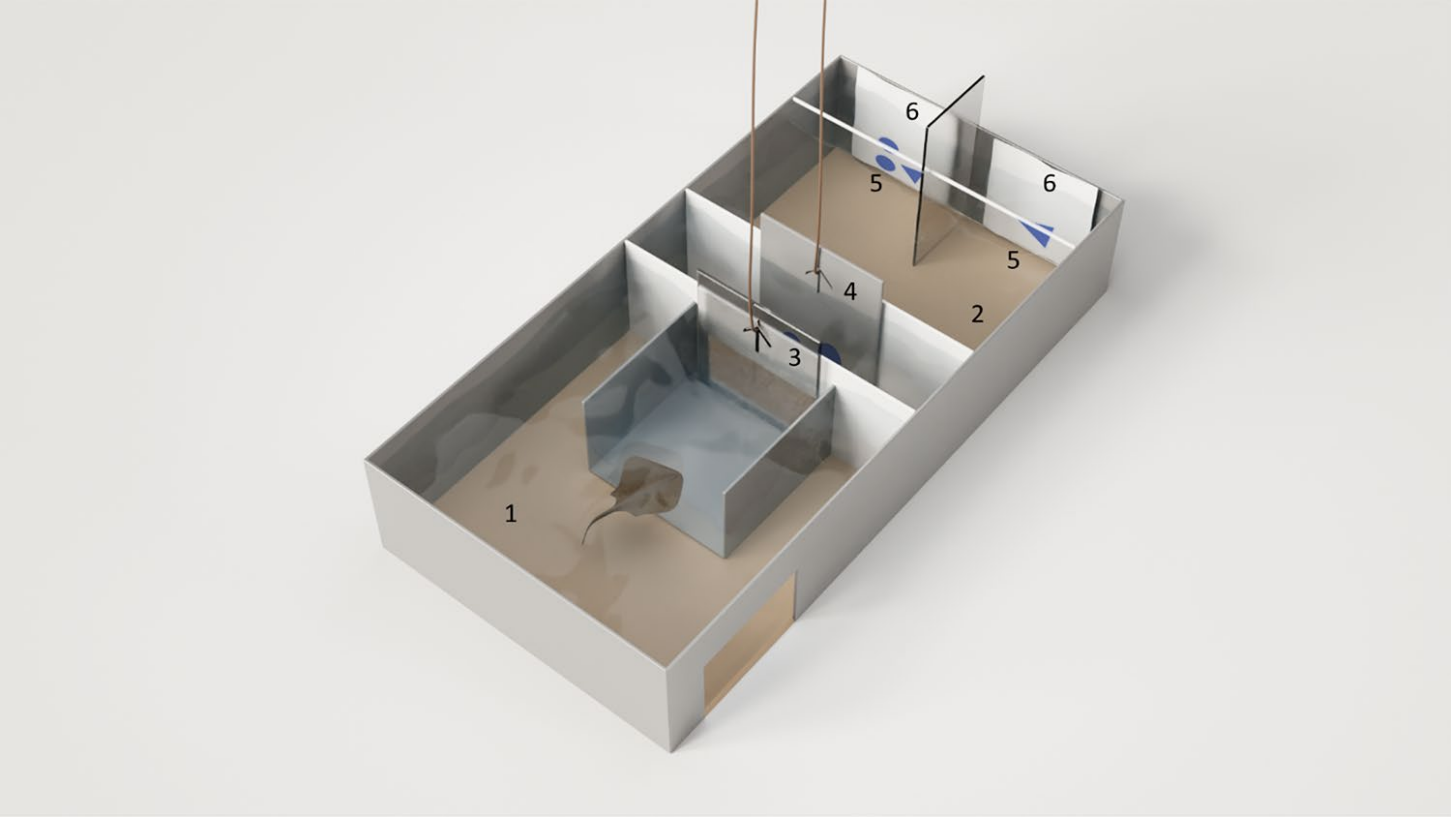
Experimental setup for the stingrays. 1) start box, 2) experimental area, 3) guillotine door, 4) door with test stimulus, 5) decision areas, 6) choice stimulus cards.

All stingrays were kept together in 1300 L of water in a single 2.30 m × 2.07 m × 0.40 m holding tank that also contained the experimental apparatus (Fig. 2). The experimental set-up (53.5 cm × 106 cm) consisted of a starting compartment (SC, including a start box (SB)), a transition area, and a decision area, separated by two manually operated guillotine doors. Stimuli were shown using laminated cards (A4) on the front side of the tank, which was separated by a plastic divider into two decision areas. Following a correct choice, food was provided via forceps. When not engaged in trials, stingrays could freely swim throughout the entire setup, whereas during experiments the experimental arena was closed off. Temperature was maintained between 27° and 29 °C and conductivity between 390 and 420 μS. Water was filtered at a constant rate of 270 L/h and partially exchanged once per week to keep nitrite values below 0 0.05 mg/L. Light intensity in the experimental room was 320 lx. All tests were observed via a Logitec camera, which was placed on a plastic rope above the set-up.

### General training procedure

All experimentally naïve fish were first accustomed to the experimental procedure by feeding them from a pipette (cichlids) or forceps (stingrays). This was followed by training them to swim through the guillotine doors towards the decision area to search for food along the stimulus wall, i.e., at the milky-colored front onto which a white PowerPoint slide was projected (cichlids) or in front of two blank stimulus cards (stingrays). If the fish swam successfully through the gates to feed from the pipette/forceps without fear, experiments commenced.

Training consisted of a ‘delayed matching-to-sample experiment’, in which a test stimulus was shown first (then removed), followed by the presentation of two choice stimuli (stimulus A and B), one of which was associated with a food reward while the other was not. Test and choice stimuli varied in how they were presented on the slides and cards, e.g. in regards to surface area, size and arrangement of stimuli (total area covered), to control for any confounding variables and reduce learning to using numerical differences only. At least ten randomly used versions existed for each number per color.

Two daily sessions (at least five hours apart) were conducted six days per week, with ten trials per session. Stimuli were presented in a pseudo-random order, appearing equally often on either side of the divider on the front wall but never more than twice consecutively on the same side. There was an inter-trial-interval (ITI) of 30 s. If a fish did not choose a stimulus within one minute, the trial was aborted; three aborted trials terminated a session. Training for each individual was considered successful or completed as soon as a learning criterion (LC) of ≥ 70% correct choices in three consecutive sessions was reached (χ2 (1) ≤ 0.05; to show statistical significance).

After successful training, fish entered the overtraining phase in which transfer tests were conducted. In this phase, a session consisted of ten regular trials (as during training) and two to three additional transfer test trials, which were randomly interspersed with regular ones during a session. To prepare the fish for transfer test trials, which were always unrewarded, food was only provided in eight out of ten correct (regular) trials in any sessions following successful training. This could lead to a drop in performance by the individuals once the LC was achieved. Prior to each session, it was randomly determined which trials remained unrewarded (regardless of the actual choice made by the fish). The first and last trials within a session were always rewarded and there were never two unrewarded trials in a row. Not rewarding some of the regular trials was intended to prevent fish from realizing that transfer trials were unrewarded and, therefore, not worth participating in.

#### Cichlids

First, while still in the starting compartment, cichlids were presented with the original test stimulus for five seconds on the milky-colored front of the tank. Cichlids observed this stimulus through the closed but transparent guillotine door. After five seconds, the stimulus presentation changed to the two choice stimuli and the door was opened. As soon as the test animal swam through the gate (crossed the line with its mouth), time (in seconds) was taken. The moment the animal crossed the decision line with its mouth, time was stopped and choice recorded. If the animal made a correct choice, it was rewarded, if the choice was incorrect, the projection was stopped and a black slide was shown instead. The animal was then led back into the starting compartment, the gate was closed and (if necessary) the tube was equipped with a new pellet.

#### Stingrays

The first guillotine door was raised and stingrays were presented with the original test stimulus for five seconds (Fig. 2). Then, the second guillotine door was raised and stingrays could enter the experimental area and the decision area, where the two choice stimuli were presented on laminated cards on either side of a divider. As soon as the stingray swam through the second gate (crossing the line with its disc), time was taken. The moment the animal crossed the decision line with its disc, time was stopped and choice recorded. If the animal made a correct choice, it was rewarded, if the choice was incorrect, cards were removed. The animal was then guided back into the starting compartment and the gates were closed.

### Stimuli

**Figure 3:**
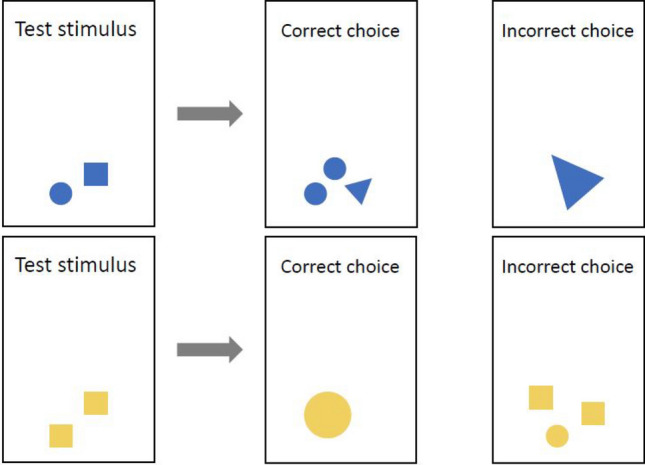
Example for a test stimulus and the corresponding two choice stimuli during addition (blue) and subtraction (yellow).

Similar to the study by Howard et al.^53^, it was tested whether individuals of both species can learn to recognize colors as symbols for addition (blue) and subtraction (yellow) by the factor ‘one’ and to identify the correct result of the respective arithmetic problem. First, fish were presented with the test stimulus for five seconds, featuring a specific set of geometric symbols (square, circle, triangle) in either yellow (two, four or five) or blue (one, two or four). Choice stimuli then depicted numbers of symbols plus or minus one object (Fig. 3). In the blue addition tasks, the fish had to select the stimulus that featured one element more than the test stimulus. In the yellow subtraction tasks, the fish had to select the stimulus that featured one symbol less (Fig. 3). The number three was not shown as an initial stimulus during training but only used in subsequent transfer tests. The total area of ​​the symbols shown within one ‘stimulus’ was 36 cm^2^. For one symbol this amounted to 36 cm^2^, for each of the two symbols to 18 cm^2^ and so on. As in other experiments, the test animals had to complete two sessions of ten trials each day, in which they each had to solve five addition and five subtraction tasks. Stimulus cards (stingrays) were laminated and measured 21.0 × 29.7 cm in size (A4).

After successful training, two to three transfer test trials were randomly interspersed with the ten regular trials per session. There were three types of transfer tests (A–C), as explained in the following section. A total of 20 trials per type of transfer test were conducted per fish. Transfer tests were unrewarded; they were neither conducted in the very beginning nor at the end of a session, and never preceded or followed one of the unrewarded regular trials. In each session, the tested animals had to complete five regular addition and subtraction tasks. The 80% reward was continued in the transfer phase. Each stimulus was presented equally often on the right and left side in the transfer tests. There were no correct or wrong choices in any of the transfer tests, choices were simple reflecting and indicative of what strategies had been used during training to solve the task. Nonetheless, any choices referring to the expected decision (having learned to add or subtract one based on the color presented) are referred to from now on as ‘correct’, while other choices are considered ‘wrong’.

### Transfer test type A

#### (Test stimulus: 3) (Addition (blue): 4 (correct choice); 2 (wrong choice)) (Subtraction (yellow): 2 (correct choice); 4 (wrong choice))

In this transfer test, the previously unused number of three symbols was presented as test stimulus (to rule out symbol memorization) and fish had to then choose between two and four symbols as choice stimuli. This transfer test was performed using both colors. When the blue symbols were presented, it was expected that fish would select the higher object number, i.e., add ‘one’. For yellow symbols, on the other hand, it was expected that the fish would subtract ‘one’.

### Transfer test type B

#### (Test stimulus: 3) (Addition (blue): 4 (correct choice); 5 (wrong choice)) (Subtraction (yellow): 2 (correct choice); 1 (wrong choice))

This test determined whether fish had learned to select the stimulus featuring the highest or lowest number of objects available based on the color presented or whether fish had actually learned to add or subtract ‘one’ from the test stimulus. The choice stimuli in this transfer test featured either plus one and plus two (blue) or minus one and minus two (yellow).

### Transfer test type C

#### (Test stimulus: 3) (Addition (blue): Adjusted stimuli size; 4 (correct choice); 2 (wrong choice)) (Subtraction (yellow): Adjusted stimuli size; 2 (correct choice); 4 (wrong choice))

This transfer test controlled for the influence of the only potentially confounding factor, i.e., individual symbol size. While surface shape, arrangement and area covered of stimuli were controlled for on the many experimental stimulus cards/projections, individual symbol size was to a lesser extent (just on some cards). This resulted in individual symbols being larger for lower numbers and smaller for higher numbers most of the time. To control for this bias, the relationship was reversed, with the individual symbols for the lower option now being smaller and the ones for the higher option now being larger.

### Data analysis

We applied generalized linear mixed models (GLMM) in R (4.0.3), using a binomial distribution with success as the response variable (0 = success, 1 = failure), the test stimulus as fixed effect and the individual as random effect. Individual was included as random effect to account for correlations that can arise through performance differences between individuals. For each group and transfer test separate models were used, as well as for addition and subtraction. *P* ≤ 0.05 was considered significant for all tests and *p* ≤ 0.001 highly significant.

### Ethics statement

The research reported here was performed under the guidelines established by the current German animal protection law. The experimental protocol for behavioral trials on fish was approved by the ethics committee of the LANUV (State Office for Nature, Environment and Consumer Protection North Rhine-Westphalia, Germany; AZ 81-02.04.2020.A432). All applicable international, national, and/or institutional guidelines for the care and use of animals were followed. The study was carried out in compliance with the ARRIVE guidelines.

## Results

### Cichlids

#### Addition/subtraction training

**Figure 4:**
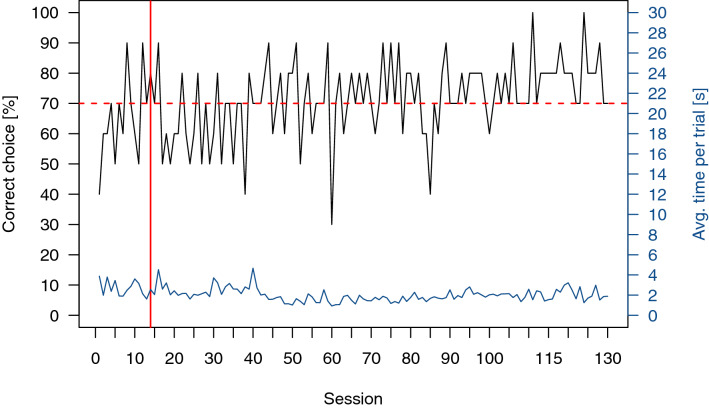
Exemplary graph of the performance of a cichlid during the entire training and testing (overtraining) phase. The black line shows the percentage of correct choices per ten-trial session. The horizontal red dotted line indicates the time the LC was reached, while the vertical red line indicates when the LC was achieved. In the sessions following the red vertical line, transfer tests were added to the regular ten trial session. The blue line gives the average trial time for each session.

All six individuals participating in the training reached the LC on average in 28 ± 18 Sessions and participated in all subsequent transfer tests. An exemplary graph of the performance throughout the whole experimental time of one cichlid is shown in Fig. 4.

#### Transfer tests

**Figure 5:**
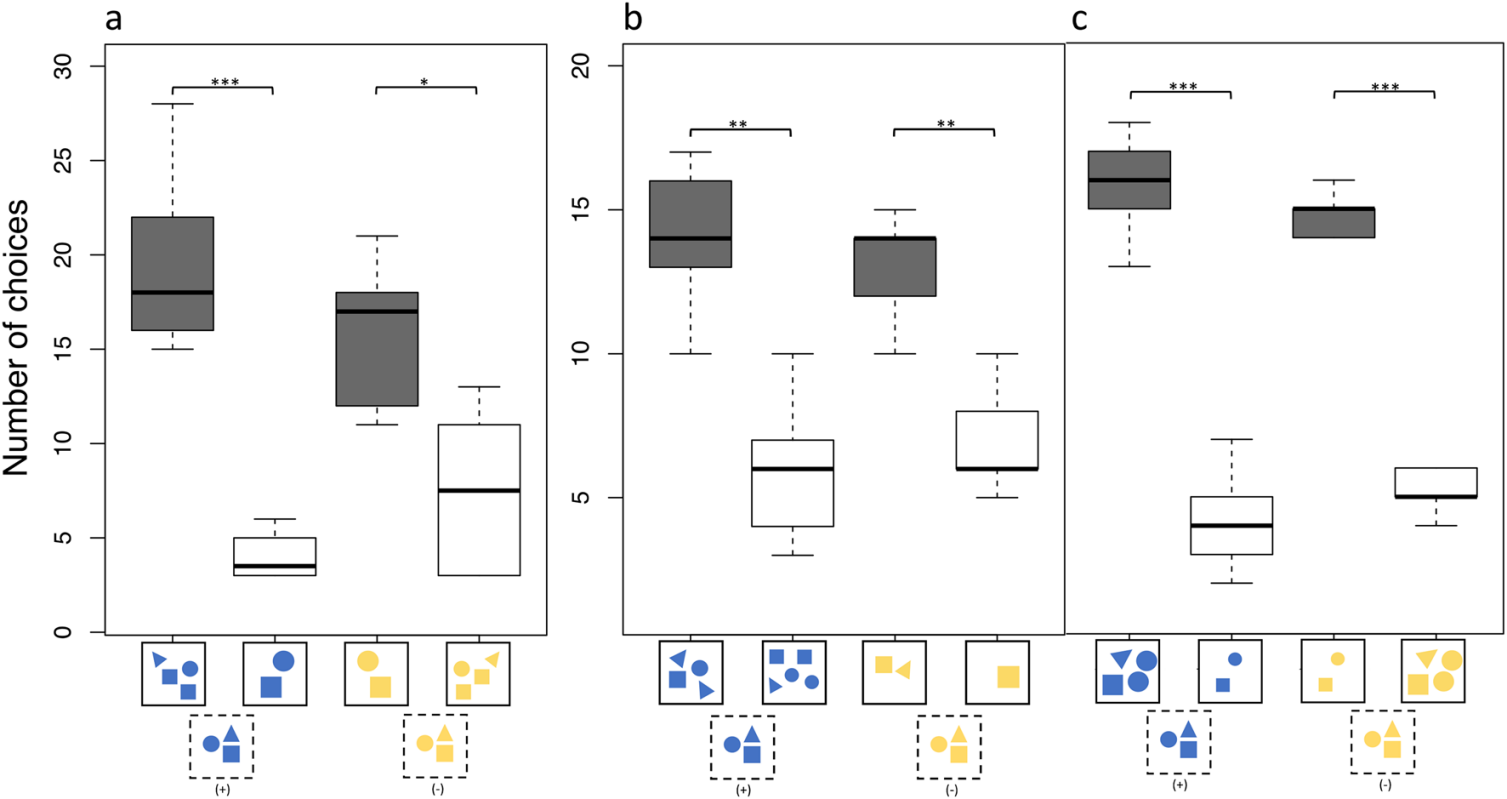
Overview of cichlid (n = 6) choices in all three transfer tests. (**a**) Transfer test type A; (**b**) Transfer test type B; (**c**) Transfer test type C. Schematic examples on which the individuals based their choice are presented on the x-axis. Stimuli in the dashed box show the test stimulus presented first, and solid boxes contain the choice stimuli that followed. Dark bars represent correct choices, transparent bars incorrect ones. *p* < 0.01*, *p* < 0.001**, *p* < 0.0001***

During transfer tests A, B and C, individual choices pooled as a group showed that the cichlids chose significantly often the correct stimulus (+ 1/− 1). Following an experimental restructuring, three individuals received more than 20 trials for transfer test A (see table in supplementary material). In the addition task of transfer test A individuals were correct in 117 out of 141 trials (n = 6, *p* < 0.0001) and 96 out of 141 times (n = 6, *p* < 0.01) in the subtraction task (Fig. 5a). In transfer test B individuals were correct 84 out of 120 times in the addition task (n = 6, *p* < 0.001) and 79 out of 120 times (n = 6, *p* < 0.001) in the subtraction task (Fig. 5b). Choices in the addition task of transfer test C were correct 95 out of 120 times (n = 6, *p* < 0.0001) and 89 out of 120 times (n = 6, *p* < 0.0001) in the subtraction task (Fig. 5c).

### Stingrays

#### Pretraining

All rays succeeded in the pretraining on average within 21 ± 5 sessions and were permitted to participate in the Addition/Subtraction training.

#### Addition/subtraction training

**Figure 6:**
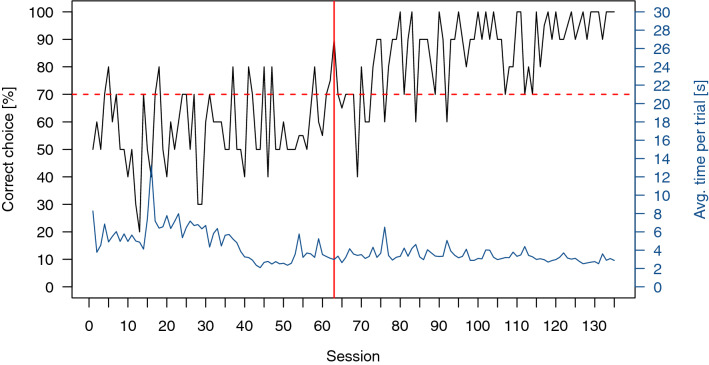
Exemplary graph of the performance of a stingray during the entire training and testing (overtraining) phase. The black line shows the percentage of correct choices per ten-trial session. The horizontal red dotted line indicates the lower limit of the LC, while the vertical red line indicates the time when the LC was achieved. In the sessions following the red vertical line, transfer tests were added to the regular ten trial session. The blue line gives the average trial time for each session.

Only four out of eight individuals reached the LC in the training. It took on average 68 ± 10 sessions for an individual to reach the LC. However, one individual could not keep up its performance and was therefore excluded from transfer tests. The remaining three individuals participated in all transfer tests. An exemplary graph of the performance of one ray throughout the whole experimental period can be seen in Fig. 6.

#### Transfer tests

**Figure 7:**
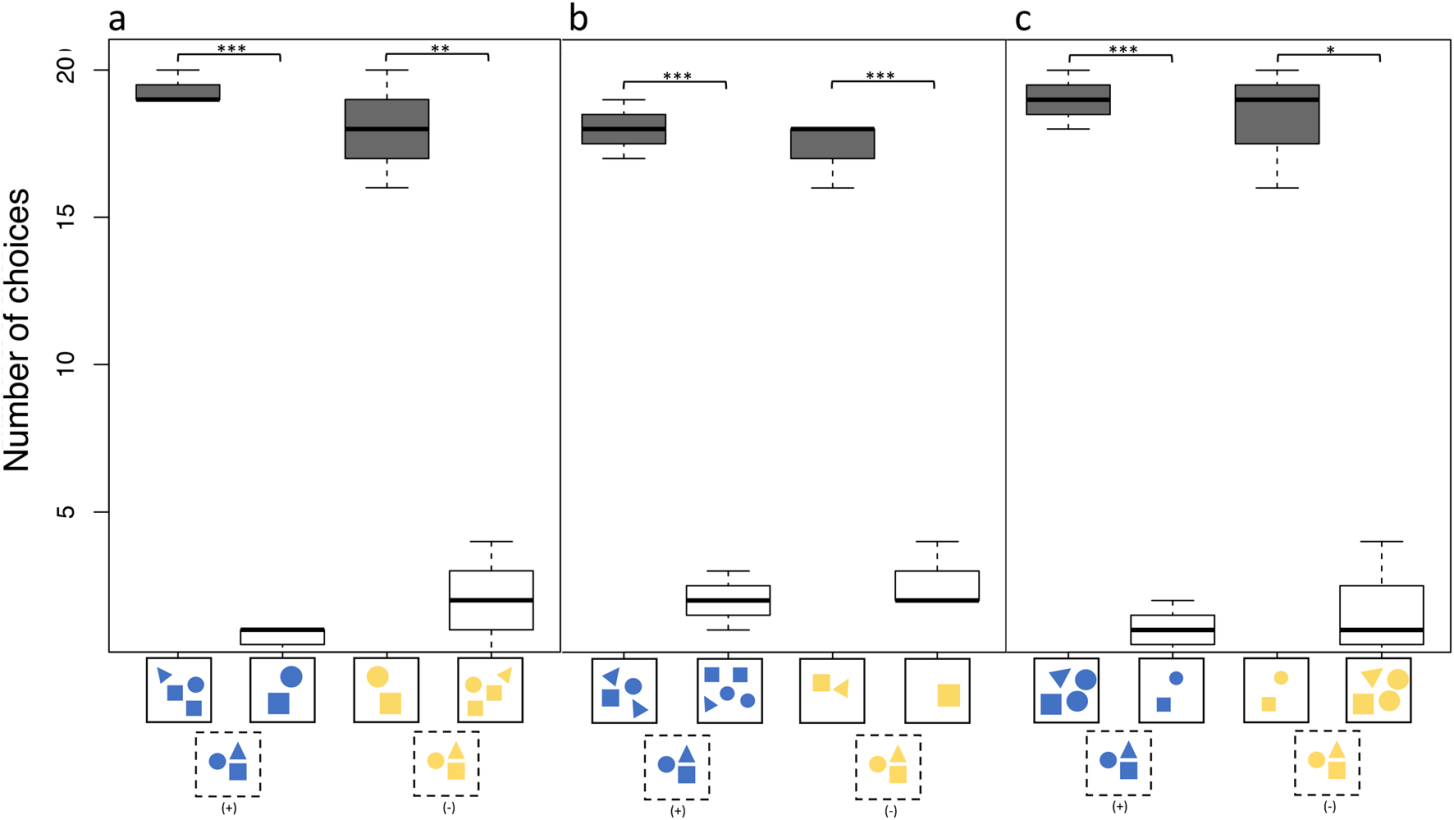
Overview of stingray (n = 3) choices in all three transfer tests. (**a**) Transfer test type A; (**b**) Transfer test type B; (**c**) Transfer test type C. Schematic examples on which the individuals based their choice are presented on the x-axis. Stimuli in the dashed box show the test stimulus presented first, and solid boxes contain the choice stimuli that followed. Dark bars represent correct choices, transparent bars incorrect ones. *p* < 0.01*, *p* < 0.001**, *p* < 0.0001***

During transfer tests A, B and C, individual choices pooled as a group showed that the stingrays chose significantly often the correct stimulus (+ 1/− 1). In the addition task of transfer test A individuals were correct 58 out of 60 times (n = 3, *p* < 0.0001) and 54 out of 60 times (n = 3, *p* < 0.001) in the subtraction task (Fig. 7a). In transfer test B individuals were correct 54 out of 60 times in the addition task (n = 3, *p* < 0.0001) and 52 out of 60 times (n = 3, *p* < 0.0001) in the subtraction task (Fig. 7b). Choices in the addition task of transfer test C were correct 57 out of 60 times (n = 3, *p* < 0.0001) and 55 out of 60 times (n = 3, *p* < 0.01) in the subtraction task (Fig. 7c).

## Discussion

Cichlids as well as stingrays successfully learned to complete a delayed matching to sample task in addition to performing a simple arithmetic task, depending on the color of the test stimulus first presented to them. Transfer tests showed that learning was independent of straightforward symbol memorization (Transfer test A). Individuals did not just learn to pick the highest or lowest number presented based on the respective color; instead, learning was specific to adding or subtracting ‘one’ (Transfer test B). While group results were significant for both species in all transfer tests, individual results varied (see supplementary information). Generally, addition was learned more easily than subtraction by both species. Fewer sessions were needed and general performance in the regular trials of the training and overtraining phase was higher. Cichlids learned the original task faster than stingrays, which may be attributed to a higher ecological relevance or to the fact that cichlids were not naïve at the start of the experiments, having participated in other cognition experiments previously. Additionally, more cichlids (80%) than stingrays (50%) learned the task. On the contrary, individual performance of stingrays generally exceeded that of cichlids, with more individuals performing significantly above chance level in all individual test scenarios.

While the outcome is not really surprising given the previous cognition tasks mastered by fish, the results describe a new level of cognitive ability in these species nonetheless. A delayed matching to sample task was paired with an arithmetic task, requiring both short-term memory, numerical competency and the combined usage of both, depending on a visual discrimination task (differentiating between two colors). Comparisons to previous studies, including the study on honeybees^53^ are difficult, due to the different training regimes. For example, the current study was solely based on a reward system while the honeybees were both rewarded and punished for their respective choices. A more interesting question—similar to the one outlined in Howard et al.^53^, as to how large a brain really needs to be to perform complex cognitive feats (e.g. considering the tasks bees succeeded in)—is as to why animals such as fish, who are lacking a neocortex or an equally structured and in many respects homologous avian pallium^63,64^, are still commonly referred to as ‘primitive’ or ‘lower’ vertebrates. Obviously, a (neo-)cortex as such is not needed to perform complex cognitive skills; instead, several studies have shown various pallial structures to be involved in selected cognitive activities in fish^4,65–67^, but there may also be others. It seems obvious, that fish, their cognitive skills and their status to be considered as sentient animals urgently needs to be revisited, specifically in light of the detrimental anthropogenic threats fish face every day.

The good results obtained in this study are somewhat surprising, when considering that the available ecological information on the species, as well as results of previous cognition studies indicate, that the two species do not have an obvious ecological or behavioral need for excelling in numerical tasks, let alone in arithmetic processes^27–29,68–70^. Both are opportunistic feeders not hunters, that show no mating- or reproduction related behaviors relying on numbers (e.g. counting stripes or eggs). Neither species nests nor is there any information available about preferences for particular sized social groups. Nonetheless, there may still be important yet so far unknown behaviors in both species relying on quantitative skills, including numerical competency. Arithmetic abilities could be one of many cognitive byproducts that may be useful to enhance individual recognition (e.g. by using phenotypic characteristics) or help detect changing environmental or socials conditions. As both species live in complex habitats (rocky lake and coral reef environments), a certain degree of behavioral flexibility is essential for survival^71–73^. To possess an enhanced cognitive skill might be advantageous under some environmental circumstances but not possessing it might not necessarily present a disadvantage either. Within this study, only three out of eight stingrays and six out of eight cichlids learned the task and took part in transfer tests. Comparable or even lower numbers of individuals were found to be successful in related numerical tasks in several other studies^27–29,70^. Despite this potential lack in needing numerical competency for natural every-day life applications, successful fish showed abilities far above chance level, specifically in the stingrays. Again, this raises the question of what abilities fish may be capable of if being asked the ‘right’ question, i.e., asked to solve an ecologically relevant question. Results also reflect on the large intraspecific cognitive and behavioral breadth found in a group (the fishes) that is rarely associated with personality or the presence of individual cognitive variation. Not only do individual differences seem to play an important role in these kind of discrimination tasks, as reported before, but possibly the origin of individuals. Stingrays examined by Kreuter et al. were not able to differentiate between 4 and 5 while the individuals in the present study showed high performance in relation to this task^27^. Individuals from this study originated from Frankfurt Zoo while the ones trained by Kreuter et al. came from Antwerp Zoo^27^.

While all group results were significant, results showed that some individuals, specifically in *P. zebra*, had more problems solving the subtraction than the addition tasks. This was not observed in the honeybees^53^. A possible reason could be the prior exposure of these individuals to other numerical tasks, in which fish frequently had to choose the larger of two amounts, while never having been exposed to a subtraction task before. Another explanation could be that under natural conditions, animals are generally more likely to choose the larger of two samples, such as the more abundant food source or the larger social group. Despite this potential preference for choosing the highest numerical stimulus, and thereby possibly representing the most surprising results of all, transfer test B showed that all of the stingrays and some of the cichlids did not simply learn to always pick the largest (or smallest) numerical value presented to them, but in fact learned to pick the stimulus differing from the sample stimulus by the factor one exactly. There really was no correct or wrong answer to the trials in transfer test B, as training never specified that stimuli had to differ from the sample stimulus by one (i.e., there was just no alternative to a difference of one and thus no punishment or missed reward for choosing something else). Had animals merely learned during training to pick the largest or the smallest number of objects provided, the transfer test results would have been very different than observed, i.e., ± 2 would have been chosen most often. Choosing ± 1 over ± 2 shows that fish paid more attention to the detailed aspects of the task than would have theoretically been necessary to be successful during training. Results thereby demonstrate once again, that even artificially created tasks, unlikely to be relevant—and possibly even unfavorable—in nature, can still be learned and applied by fish. Overall, it seems likely that fish, independent of whether there is a direct biological need or not, can solve complex numerical tasks.

The current study only assessed arithmetic abilities within the range of the OFS (and up to a ratio of 0.75), which is similar to other animals tested^74–76^. Experiments assessing 5:10 (ratio 0.5) and 15:20 (ratio 0.75) in cichlids were only successful in very few or no individuals, respectively^29,77^. Stingrays were successfully tested in 8:12 (ratio 0.67) but were unable to perform 9:12 (ratio 0.75)^27^, a finding that matches those for guppies, zebrafish, redtail splitfin, and Siamese fighting fish^78^. Therefore, it is unlikely that fish can extrapolate similar arithmetic tasks as performed here (e.g., ± 5) onto numbers residing outside of the OFS, even if the ratio presented is similar or lower (i.e., the difference greater) than found suitable for differentiation within the OFS.

In conclusion, the ability to ‘count’ and to perform simple arithmetic processes is not just present in humans, non-human primates and birds^41–49^, but also in invertebrates such as honey bees and spiders^51–53^ and not surprisingly also in fish, both teleosts and elasmobranchs. Large intraspecific differences (cichlids) and a considerably high number of unsuccessful individuals (stingrays) indicate that numerical abilities may not be of particular importance to both *P. zebra* and *P. motoro*. Nonetheless, individuals that passed the training stages maintained very high-performance levels. Results confirm previous findings that fish possess many of the same cognitive abilities and to a similar extent as birds and mammals.

## Supplementary Information

Supplementary Information.
