# Supplementary material for: Cichlids and stingrays can add and subtract ‘one’ in the number space from one to five
Source: Sci Rep. 2022 Mar 31;12:3894. doi: 10.1038/s41598-022-07552-2 (PMC8971382; doi:10.1038/s41598-022-07552-2)
Supplement: Supplementary file 1 — Supplementary Information. [file 41598_2022_7552_MOESM1_ESM.pdf]

Overview of individual performances for each transfer test

	Transfer test A		Transfer test B		Transfer test C	
**Cichlids**	**add**	**sub**	**add**	**sub**	**add**	**sub**
Individual 1	15/20<br>$p<0.05$	12/20<br>n.s	17/20<br>$p<0.01$	10/20<br>n.s	16/20<br>$p<0.05$	14/20<br>n.s
Individual 2	28/31<br>$p<0.0001$	18/31<br>n.s	14/20<br>n.s	14/20<br>n.s	17/20<br>$p<0.01$	15/20<br>$p<0.05$
Individual 3	22/28<br>$p<0.01$	21/28<br>$p<0.05$	10/20<br>n.s	14/20<br>n.s	16/20<br>$p<0.05$	15/20<br>$p<0.05$
Individual 4	19/22<br>$p<0.001$	11/22<br>n.s	14/20<br>n.s	14/20<br>n.s	13/20<br>n.s	15/20<br>$p<0.05$
Individual 5	16/20<br>$p<0.05$	17/20<br>$p<0.01$	16/20<br>$p<0.05$	12/20<br>n.s	18/20<br>$p<0.001$	14/20<br>n.s
Individual 6	17/20<br>$p<0.01$	17/20<br>$p<0.01$	13/20<br>n.s	15/20<br>$p<0.05$	15/20<br>$p<0.05$	16/20<br>$p<0.05$
**Stingrays**						
Individual 1	19/20<br>$p<0.0001$	20/20<br>$p<0.0001$	17/20<br>$p<0.01$	18/20<br>$p<0.001$	20/20<br>$p<0.0001$	20/20<br>$p<0.0001$
Individual 2	20/20<br>$p<0.0001$	18/20<br>$p<0.001$	19/20<br>$p<0.0001$	18/20<br>$p<0.001$	18/20<br>$p<0.001$	19/20<br>$p<0.0001$
Individual 3	19/20<br>$p<0.0001$	16/20<br>$p<0.05$	18/20<br>$p<0.001$	16/20<br>$p<0.05$	19/20<br>$p<0.0001$	16/20<br>$p<0.05$

Supplementary table. Individual performances for each species during the transfer tests. Green indicates significant and red non significant performance. Correct choices are shown in relation to total number of trials.
